# Enhancing plasmid typing with MOB-typer: resolving IncP and other incompatibility group misclassifications in Pseudomonas

**DOI:** 10.1099/mgen.0.001491

**Published:** 2025-09-09

**Authors:** Masato Suzuki, Haruo Suzuki, Yosuke Nishimura, Hideaki Nojiri, Masaki Shintani

**Affiliations:** 1 Antimicrobial Resistance Research Center, National Institute of Infectious Diseases, Japan Institute for Health Security, 4-2-1 Aobacho, Higashimurayama, Tokyo 189-0002, Japan; 2 Faculty of Environment and Information Studies, Keio University, 5322 Endo, Fujisawa, Kanagawa 252-0882, Japan; 3 Research Center for Bioscience and Nanoscience (CeBN), Japan Agency for Marine-Earth Science and Technology (JAMSTEC), 2-15 Natsushima-cho, Yokosuka-city, Kanagawa 237-0061, Japan; 4 Agro-Biotechnology Research Center (AgTECH), Graduate School of Agricultural and Life Sciences, The University of Tokyo, 1-1-1 Yayoi, Bunkyo-ku, Tokyo 113-8657, Japan; 5 Department of Engineering, Graduate School of Integrated Science and Technology, Shizuoka University, 3-5-1 Johoku, Chuo-ku, Hamamatsu, Shizuoka 432-8561, Japan; 6 Research Institute of Green Science and Technology, Shizuoka University, 3-5-1, Johoku, Chuo-ku, Hamamatsu, Shizuoka 432-8561, Japan; 7 Japan Collection of Microorganisms, RIKEN BioResource Research Center, 3-1-1 Koyadai, Tsukuba, Ibaraki 305-0074, Japan

**Keywords:** IncP, IncP-1 to P-14, Plasmid, *Pseudomonas*, replicon typing

Plasmids are mobile genetic elements that facilitate horizontal gene transfer (HGT) among diverse bacteria [1]. By carrying accessory genes, including those involved in antimicrobial resistance (AMR) and/or metabolism of various compounds, plasmids promote rapid bacterial evolution and adaptation to natural and host-associated environments. Plasmids are major contributors to the emergence and spread of antimicrobial-resistant bacteria, posing a global public health threat [2]. AMR genes are frequently carried on plasmids and spread among bacteria through conjugation. The precise classification of AMR plasmids is crucial for genomic epidemiological studies, particularly in clinically relevant bacterial pathogens, such as *Pseudomonas aeruginosa*.

The MOB-suite tools, introduced in two influential studies published in 2018 and 2020 [3,4], have become indispensable in plasmid research. These tools, particularly MOB-typer, have significantly advanced the identification of plasmid types and conjugative properties by analysing replication-associated genes, including replication initiator protein (RIP) genes (replicon types), relaxase genes (MOB classes) and type IV secretion system genes (mating pair formation families). They have provided researchers with essential capabilities to explore plasmid diversity, predict host ranges and conduct sequence-based studies. The widespread adoption of MOB-typer in studies [5,7] and its integration into databases, such as the updated PLSDB [8], highlight its impact and usability in the scientific community. However, we have identified systematic misclassification issues in MOB-typer, particularly regarding the accurate identification and classification of *Pseudomonas* plasmids.

Historically, plasmids have been classified based on incompatibility (Inc) groups, a method that relies on the inability of two plasmids from the same group to coexist in a single host cell due to similar replication or maintenance systems [9]. As model organisms in plasmid research, *P. aeruginosa* and *Escherichia coli* have traditionally been studied independently, each contributing to the development of distinct Inc group classification systems. In *Pseudomonas*, experimental studies since the 1970s have defined Inc groups ranging from IncP-1 to IncP-14 [10]. In particular, the IncP-1 group also served as the basis for defining the concept of a conserved plasmid backbone, which has become central to understanding plasmid modularity and evolution [11]. Similarly, within the order *Enterobacterales*, Inc groups from IncA to IncZ have been defined based on their biological features [12]. Notably, the same broad-host-range plasmid can be classified into different Inc groups depending on the bacterial host. For instance, IncP-1 in *Pseudomonas* aligns with IncP in *Enterobacterales*, IncP-3 corresponds to either IncA or IncC, IncP-4 is equivalent to IncQ and IncP-6 corresponds to IncG.

This overlapping terminology has led to confusion, particularly in how MOB-typer classifies *Pseudomonas* plasmids. MOB-typer often assigns multiple *Pseudomonas* plasmid groups to the generic label ‘IncP’. For instance, plasmid groups such as IncP-2 [13,15], IncP-6 [16,17], IncP-7 [18,21] and IncP-9 [22,24], all of which are well-documented in the literature, are incorrectly classified as ‘IncP’ (Tables 1 and S1, available in the online Supplementary Material). This mislabelling obscures the evolutionary and functional distinctions among these groups and erroneously merges them with the IncP-1 group. Although these plasmids share similar ‘IncP-’ derived names, they represent distinct replicon types with distinct evolutionary origins, biological characteristics and replication-related nt/protein sequences and domain architectures.

**Table 1.** Bacterial plasmids detectable with MOB-typer and PlasmidFinder

Plasmid typing tool^*^	No. of sequences	*Pseudomonas*	*Enterobacterales*	Reference
MOB-typer v3.1.9	2,685	Some of IncP (=IncP-1), IncA/C (=IncP-3), IncQ (=IncP-4), IncP-6 (=IncG), IncP-7 and IncP-9 plasmids	IncA to IncZ plasmids and Col plasmids	[3,4]
PlasmidFinder v2.1	488	Some of IncP (=IncP-1), IncA/C (=IncP-3), IncQ (=IncP-4) and IncP-6 (=IncG) plasmids	IncA to IncZ plasmids and Col plasmids	[28,29]

*The database versions are v3.1.9 (as of 4 June 2024) for MOB-typer and v2.1 (as of 19 April 2025) for PlasmidFinder.

The number of sequences and the target plasmids in *Pseudomonas*, *Enterobacterales* and other bacteria in their respective replicon sequence databases are shown. The number 2,685 refers to unique replicon sequences in the MOB-typer v3.1.9 database, excluding 1 redundant entry.

In the MOB-typer database, IncP-derived names, such as IncP-6, IncP-7 and IncP-9, are mislabelled as ‘IncP’.

Further complexity arises from the use of Greek-letter suffixes such as IncP*α*, IncP*β* and IncP*γ*, which denote subgroups within the IncP(IncP-1) incompatibility group based on phylogeny and sequence divergence [25,27]. In contrast, numeric designations like IncP-2, IncP-6 and IncP-9 refer to entirely separate incompatibility groups with distinct evolutionary origins and replication systems. This dual use of similar naming formats often causes confusion. For example, IncP*β* plasmids, though part of the IncP (IncP-1) group, are sometimes misidentified as other ‘IncP-’ -derived plasmids (such as IncP-2) outside this group due to their similar naming. In contrast, despite the apparent similarity in name, IncP-2 plasmids belong to a completely separate incompatibility group with distinct evolutionary origins and different replication system. Properly understood, Greek letters are meant to indicate subgroups within IncP (IncP-1), whereas numerical labels refer to unrelated ‘IncP-’-derived groups. The issue is compounded when MOB-typer labels all of them generically as ‘IncP’. Clarifying both naming schemes is essential for accurate and consistent plasmid classification.

Such misclassification not only misrepresents plasmid diversity but also risks causing significant confusion in the research field. Researchers relying on MOB-typer for plasmid classification may inadvertently propagate inaccuracies in their publications, leading to downstream errors in plasmid-based studies and applied research, such as genomic epidemiological studies of AMR, which rely on robust and cumulative fundamental knowledge. Given the importance of accurate plasmid classification for understanding HGT, AMR and plasmid–host interactions, addressing these issues is essential.

The impact of these misclassifications extends to widely used plasmid databases where the integration of MOB-typer has introduced replicon-level errors that affect plasmid annotation and classification. A prominent case is the PLSDB, a curated and searchable collection of complete plasmid sequences derived from publicly available bacterial genomes [8,28, 29]. The latest updates to PLSDB in October 2024 incorporated MOB-typer for plasmid typing, inadvertently introducing classification errors into the database [26]. In the PLSDB release (v2024_05_31_v2), a total of 656 plasmids were labelled as ‘IncP’. Of these, at least 234 plasmids were found to belong to distinct *Pseudomonas* plasmid groups, such as IncP-2, IncP-3, IncP-4, IncP-6, IncP-7, IncP-9 or their hybrids (Table S2).

MOB-typer’s replicon sequence database (consisting of 2,685 sequences in v3.1.9, as of 4 June 2024) was originally developed as an extension of the PlasmidFinder database (Table 1) [3,4, 28, 29]. However, replicon group names, such as IncP-1 and IncP-6, which are preserved in PlasmidFinder, have been lost in MOB-typer, where they are collectively referred to simply as ‘IncP’ (Table S1). Recognizing the significance of this issue, we contacted the authors of the PLSDB publications directly to discuss potential solutions. In response, they kindly agreed to reintegrate PlasmidFinder as an additional plasmid typing tool, in January 2025, in order to enhance annotation accuracy. This proactive response underscores the importance of community feedback in maintaining the reliability of shared resources.

Moreover, the MOB-typer database includes a sequence derived from the IncP-2 plasmid pOZ176 (accession number NC_022344), which corresponds to an auxiliary RIP gene and is listed as ‘IncP’ (‘IncP’ with NC_022344 in Table S1), despite clear evidence from our study reported in 2022 that it is not the primary RIP (*repP-2A*) gene of the IncP-2 plasmid [13]. As a result, it is misclassified in two ways – first, by being incorrectly identified as the primary RIP gene in *Pseudomonas*, and second, by being generically labelled as ‘IncP’ in MOB-typer. It should be noted, however, that some authentic IncP-2 plasmids carrying the *repP-2A* gene may also carry this auxiliary RIP gene. In such cases, their classification as IncP-2 remains valid. In practice, MOB-typer detected a number of plasmids in PLSDB as ‘IncP’ based on this auxiliary RIP gene, although only 16 out of 97 are valid IncP-2 plasmids (Table S2). Thus, this underscores the need for careful interpretation of replicon typing results when auxiliary RIP gene(s) are present. To facilitate correct interpretation, we have provided the precise classification of *Pseudomonas* Inc groups other than IncP-1, such as IncP-2, IncP-6, IncP-7, IncP-9 and their hybrids, among PLSDB plasmids labelled as ‘IncP’ (Table S2).

Another example involves the Comprehensive Plasmid Annotation and Sequence Search (COMPASS) database, published in 2020, a plasmid resource that provides systematically annotated complete plasmid sequences and supports comparative analyses across bacterial species [30]. Mislabelling resulting from inaccurate replicon typing by MOB-typer in this database further illustrates the risks of propagating incorrect plasmid classifications. Additionally, the failure to distinguish between plasmid classifications derived from *Pseudomonas* and those from *Enterobacterales* has led to further naming errors in a recent review [31]. Even when tools, such as the above PlasmidFinder [32,33], which are designed to correctly assign replicon types, are employed, plasmids have still been erroneously labelled as ‘IncP’ due to this oversight [31].

These examples highlight the persistent risk of misinterpretation and the propagation of errors in plasmid classification if such discrepancies remain unaddressed. Therefore, an urgent reevaluation and correction of naming conventions and classification methods is essential to prevent further misunderstandings and maintain the reliability of plasmid research.

Despite these challenges, we believe that MOB-typer remains a powerful and accessible tool for plasmid research. Given the mosaic nature of plasmids, where genes involved in replication and conjugative transfer may derive from multiple types or families, MOB-typer is particularly valuable, as it can simultaneously identify and classify gene clusters associated with both processes. Its ability to analyse plasmids from genome assemblies has significantly lowered the barrier to plasmid research for researchers worldwide. Nevertheless, tools like MOB-typer must be continually refined to ensure the accuracy and reliability of the data they produce, especially as they become increasingly integrated into widely used databases and bioinformatic workflows.

By highlighting these misclassification issues, we aim to foster constructive dialogue within the research community. Enhancing MOB-typer’s naming conventions and underlying algorithms, particularly through the incorporation of data specific to the classification of *Pseudomonas* Inc groups, could further improve its utility and help prevent the propagation of errors in the field. We hope that our observations will inspire collaborative efforts to address these challenges and uphold the high standards of accuracy that are essential to plasmid research.

## Supplementary material

10.1099/mgen.0.001491 Uncited Supplementary Material 1.

## Abbreviations

AMR: antimicrobial resistance
HGT: horizontal gene transfer
RIP: replication initiator protein
